# Predictive Factors for Hearing Loss in Congenital Cytomegalovirus Infection

**DOI:** 10.3390/audiolres15010002

**Published:** 2024-12-27

**Authors:** Virginia Corazzi, Lucia Belen Musumano, Andrea Migliorelli, Laura Negossi, Chiara Bianchini, Francesco Stomeo, Stefano Pelucchi, Andrea Ciorba

**Affiliations:** ENT & Audiology Unit, Department of Neurosciences, University Hospital of Ferrara, 44124 Ferrara, Italy; lbmusumano@gmail.com (L.B.M.); mglndr1@unife.it (A.M.); laura.negossi@unife.it (L.N.); chiara.bianchini@unife.it (C.B.); stmfnc@unife.it (F.S.); pls@unife.it (S.P.); andrea.ciorba@unife.it (A.C.)

**Keywords:** congenital hearing loss, newborn hearing screening, Cytomegalovirus, congenital infection, brain imaging

## Abstract

Objectives: The present study aims to identify potential predictive factors for developing sensorineural hearing loss (SNHL) in individuals with congenital Cytomegalovirus (cCMV) infection. Methods: A retrospective study was performed on 50 subjects with cCMV infection (symptomatic and asymptomatic), followed at the Audiology Service of Sant’Anna Hospital (University Hospital of Ferrara). The following data were analyzed: the type of maternal Cytomegalovirus (CMV) infection (primary versus non-primary), time of in utero infection, systemic signs and symptoms or laboratory test anomalies due to cCMV infection, and signs and symptoms of central nervous system (CNS) involvement at birth. In particular, brain ultrasonography and encephalic magnetic resonance imaging (MRI) were evaluated, searching for possible links between imaging findings and SNHL. Results: The statistical analysis showed a significantly higher risk of developing SNHL in subjects with signs and symptoms of CNS involvement at birth (*p* = 0.009 *). The presence of brain MRI abnormalities significantly influenced the onset of SNHL in patients with symptomatic cCMV infection (*p* = 0.012 *). Brain ultrasonography, the type of maternal CMV infection, systemic signs/symptoms and laboratory test anomalies at birth, and sex resulted in nonsignificant correlations in the analysis. Conclusions: The presence of neurological symptoms at birth and of detectable abnormalities in brain MRI are predictors of SNHL developing in symptomatic cCMV infection. Further investigation on this topic is necessary.

## 1. Introduction

Cytomegalovirus (CMV) infection is among the most frequent congenital infections in the world with a prevalence of 0.15–0.51% in Italy, according to data reported by the Istituto Superiore di Sanità [1]. Congenital CMV (cCMV) infection is the most common non-genetic cause of permanent sensorineural hearing loss (SNHL) in childhood, and the most frequent infectious cause of neurological, psychomotor, and cognitive disability [2].

Cytomegalovirus (CMV) is the human herpesvirus 5 (HHV5), a DNA virus that is transmitted directly or indirectly through saliva, urine, cervical/vaginal secretions, semen, breast milk, tears, feces, and blood. Primary infection in immunocompetent individuals may be asymptomatic or paucisymptomatic, with prolonged excretion of the virus in urine. The infection may remain latent in blood mononuclear cells and bone marrow progenitors, effectively constituting a source of viral reactivation. On the other hand, non-primary infection can be caused by the reactivation of a previous CMV infection or reinfection by a different virus strain [3].

Specifically, maternal–fetal transmission due to maternal primary CMV infections occurs in the first trimester of pregnancy in 30–35% of cases and in the third trimester in 78% [4], causing symptomatic cCMV infection in 10–15% of newborns and asymptomatic cCMV infection in 85–90%. During maternal non-primary CMV infection, in utero transmission occurs in only 0.1–1.2% of cases, leading to symptomatic cCMV infection in less than 1% of newborns and to asymptomatic cCMV infection in more than 99%. More than 60% of infants with cCMV are born to mothers with non-primary CMV infection [5].

The major sequelae of CMV in symptomatic patients include SNHL (60%), cognitive deficits (45%), cerebral palsy (35%), chorioretinitis (15%), and infant death (10%). In asymptomatic forms, the incidence of SNHL is lower (7–15%); cognitive disabilities are present in few cases (2–10%), as well as chorioretinitis and cerebral palsy (1–2% and 1% of cases, respectively) [6].

SNHL represents the most common sequela of cCMV infection; it may be present at birth (21% of cases) or have a late onset, usually around 3–4 years of age (24% of cases). Hearing deficit in cCMV infection is typically unilateral or bilateral pantonal SNHL; the hearing loss could be progressive with varying degrees of severity (mild to profound). SNHL may be uni- or bilaterally fluctuating, occasionally at just a few frequencies, more often in asymptomatic than in symptomatic forms [7]. Although the frequency of hearing loss in infants born to mothers with primary and non-primary CMV infection is similar, the severity appears to be more pronounced in the former. Kabani et al. [8] confirmed the correlation between maternal primary CMV infection contracted at any gestational age and the development of hearing loss.

Currently, there are mainly two risk factors for SNHL development in cCMV infection according to the literature: (i) central nervous system (CNS) involvement, detected by brain MRI (magnetic resonance imaging) and/or brain ultrasound [9], and (ii) high levels of viral DNA in blood (viral load > 17,000 copies/mL) [10,11] and urine [10,12] at birth.

The present study aims to evaluate the presence of predictive factors for SNHL onset (at birth or late onset) in subjects with cCMV infection in a retrospective setting.

## 2. Materials and Methods

The clinical records of 50 patients with symptomatic and asymptomatic cCMV infection, followed at the Audiology service of Sant’Anna Hospital (University Hospital of Ferrara), were retrospectively evaluated from 2008 to 2023.

### 2.1. Diagnosis of CMV Infection

As previously reported [13], the diagnostic criteria adopted to identify cCMV infection were (i) the quantification of viral DNA by PCR (polymerase chain reaction) analysis in the amniotic fluid sample [14,15]; (ii) the quantification of viral DNA by PCR in neonatal urine, saliva, and blood sample within 15–21 days of life; and (iii) the quantification of viral DNA by PCR on Guthrie card [16,17]. cCMV was diagnosed whenever one or more of the abovementioned diagnostic criteria were positive.

Data on the serological detection of IgG and IgM by enzyme-linked immunosorbent assay (ELISA) technique on maternal serum during pregnancy were also collected when available [18,19]. Maternal serologic testing was considered diagnostic for primary maternal CMV infection if (i) seroconversion was established during pregnancy and (ii) low IgG avidity was established in case of maternal positivity for both IgM and IgG [18].

The definition of symptomatic versus asymptomatic cCMV infection was made according to the criteria proposed by Rawlinson et al. [20] (notably, “Moderately to severely symptomatic congenital cytomegalovirus disease”, “Mildly symptomatic congenital cytomegalovirus disease”, “Asymptomatic congenital cytomegalovirus infection with isolated sensorineural hearing loss”, and “Asymptomatic congenital cytomegalovirus infection”). For the statistical analysis, patients with moderate-to-severe symptomatic cCMV infection were grouped together with those presenting mild symptomatic cCMV infection; furthermore, asymptomatic infants were grouped together with asymptomatic ones with isolated SNHL.

### 2.2. Audiological Testing

In this study, any form of SNHL (present at birth or late onset, unilateral or bilateral, and of any severity) was included. Late-onset SNHL was considered in any patient who bilaterally passed the newborn hearing screening (NHS) and developed hearing loss after birth. NHS was performed by Otoacoustic Emissions (OAE) and/or aABR (automated auditory brainstem response) at birth in the Nursery and/or in the NICU (Neonatal Intensive Care Unit). A retest was usually performed for unilateral or bilateral REFER results within three weeks of age. In case of a confirmation of REFER result, subsequent diagnostic audiological examination was performed at the Audiology Service by performing ABR with threshold assessment, ASSR (Auditory Steady State Responses), tympanometry and acoustic reflex threshold, and audiometric tests based on patient age.

### 2.3. Clinical and Laboratory Data

Among the systemic clinical signs and symptoms present at birth and the laboratory tests related to cCMV infection, the following were considered: intrauterine growth restriction, jaundice, hepatosplenomegaly, purpura, elevated transaminase values, hyperbilirubinemia, and thrombocytopenia.

The presence of microcephaly, seizures, and/or muscle tone abnormalities at birth were instead considered among the signs/symptoms of CNS involvement.

### 2.4. Brain Imaging

Among symptomatic patients, available brain imaging (brain ultrasonography and brain MRI) was also analyzed, searching for abnormalities related to cCMV infection.

#### 2.4.1. Brain Ultrasound

As for brain ultrasound, the following anomalies were investigated: lenticulostriate vasculopathy, brain calcifications, hydrocephaly, microcephaly, germinative cysts, ventricular dilatation, holoprosencephaly, polymicrogyria, occipital cystic lesions, temporal cysts, pseudocyst, and cerebellar hypoplasia.

#### 2.4.2. Brain MRI

The findings of brain MRI included: intracerebral calcifications, ventriculomegaly, white matter abnormalities, periventricular radiolucencies, pachygyria, lissencephaly, cortical dysplasia or atrophy, cerebellar abnormalities, hippocampal dysplasia, caudothalamic or paraventricular germinolytic cysts, and corpus callosum dysgenesis. To improve the stratification of brain MRI data, the scoring system proposed by Alarcon et al. [21] was adopted (score ranges from 0: no brain anomalies to 3: extensive anomalies).

### 2.5. Exclusion Criteria

As for exclusion criteria, patients with cCMV infection concomitantly showing comorbidities and risk factors for permanent childhood hearing loss, as described by the Joint Committee in Infant Hearing (JCIH) [22], and/or with genetic mutations related to hearing loss were excluded. In addition, patients with comorbidities that could interfere with brain imaging assessment (such as syndromes with known brain anomalies and other congenital infections affecting the CNS) were excluded.

### 2.6. Ethics

This study was conducted by our institutional and national research committee ethical standards and the Declaration of Helsinki (2008). Furthermore, the present study was performed only by retrospectively evaluating the patients’ records, and this did not affect their care and management; ethics committee approval was not required.

### 2.7. Statistical Analysis

The comparison of categorical variables between groups was carried out using the chi-square (χ^2^) test or Fisher’s exact test in the case of 2 × 2 tables and expected frequencies < 5%. Univariate and multivariate logistic regression analysis was performed to calculate the risk of independent SNHL, expressed as odds ratio (OR). Values of probability of less than 0.05 were considered statistically significant. All analyses were performed by Statistical Package for the Social Sciences (SPSS) software 29.0.1 (IBM Corp., Armonk, NY, USA).

## 3. Results

### 3.1. Descriptive Analysis of the Sample

The sample consisted of 50 subjects with cCMV infection; 26 were asymptomatic (52%) and 24 were symptomatic (48%) (see Table 1 for sample features). There were no statistically significant differences between the two groups of patients (asymptomatic and symptomatic) in terms of sex (*p* = 0.786), type of maternal CMV infection (primary versus non-primary, *p* = 0.5), time of in utero infection (*p* = 0.276), and systemic signs/symptoms and/or laboratory changes found at birth related to cCMV infection (*p* = 0.182).

Furthermore, 38/50 (76%) patients developed SNHL, including 17/26 (65.4%) subjects with asymptomatic infection and 21/24 (87.5%) with symptomatic infection. Six cases had late-onset SNHL within the group of subjects with asymptomatic cCMV infection.

In total, 12 out of 24 patients with symptomatic cCMV infection (50%) had signs and symptoms of CNS involvement at birth. Table 2 shows brain imaging abnormalities (in brain ultrasonography and/or brain MRI) identified within the group of symptomatic patients. Cerebral ventricle enlargement, lenticulostriate vasculopathy, and cystic lesions were among the most frequent abnormalities detected in ultrasound image analysis; in MRI examination, the following abnormalities were the most common: white matter alterations, ventriculomegaly, and temporal lobe lesions (namely white matter abnormalities and presence of cysts and/or enlargement of the temporal horn).

### 3.2. Risk Factors for SNHL Development

Table 3 shows all the risk factors investigated for SNHL development in the sample of subjects with cCMV infection.

Subjects with signs and symptoms of CNS involvement at birth had a significantly higher risk of developing SNHL compared with infected subjects without CNS involvement on clinical examination (*p* = 0.009 *, univariate analysis *p* = 0.999). An increased risk of SNHL onset in subjects with symptomatic cCMV infection was observed with a trend of significance compared with asymptomatic subjects (*p* = 0.67, univariate analysis *p* = 0.078).

A further trend of significance was present when analyzing the time of in utero infection, as subjects infected during the first trimester of gestation were found to be at a greater risk of developing SNHL compared to those infected in the second and third trimesters (*p* = 0.087). The analysis performed by comparing subjects infected during the second and third trimesters together with those infected during the first trimester identified a stronger trend of statistical significance (*p* = 0.057). The analysis of univariate logistic regression showed a significant influence of the gestational age in which infection is transmitted on the onset of hearing loss (*p* = 0.047 * and *p* = 0.048 *).

No statistically significant difference was detected in the occurrence of SNHL between subjects infected during a maternal primary CMV infection and those affected by a maternal non-primary infection (*p* = 0.273; *p* = 0.239 at the univariate analysis). The presence of systemic signs and/or symptoms and alterations in laboratory tests at birth due to cCMV infection were not found to be a significant factor for the onset of SNHL (*p* = 0.193, *p* = 0.999 at the univariate analysis).

Multivariate analysis showed no statistical significance for the risk factors investigated. Type of cCMV infection (symptomatic vs. asymptomatic), type of maternal CMV (primary vs. non-primary), time of infection (first vs. second vs. third trimester), and presence or absence of systemic signs/symptoms and/or laboratory changes at birth due to cCMV infection were not found to significantly affect the onset of unilateral or bilateral SNHL (*p* > 0.05). Furthermore, none of these factors were found to significantly affect the onset of severe-to-profound SNHL (*p* > 0.05).

Analysis of sex showed no statistically significant influence on the onset of hearing loss (*p* = 0.614, univariate analysis *p* = 0.615), on the occurrence of uni- or bilateral hearing loss, and on the occurrence of severe-to-profound SNHL (*p* > 0.05).

In the symptomatic group, the presence of abnormalities in brain MRI was statistically significant, as subjects with MRI anomalies scoring 1, 2, and 3 according to Alarcon et al.’s classification [21] were found to be at a greater risk of developing SNHL than subjects scoring 0, i.e., without MRI brain anomalies (*p* = 0.012 *).

Moreover, the occurrence of SNHL in symptomatic patients did not correlate with the presence of abnormalities detected in brain ultrasound (*p* = 0.774).

Multivariate analysis of the risk factors related to brain imaging examination showed no statistical significance.

In the symptomatic group, abnormalities found in brain imaging (ultrasound and MRI) and the presence of signs and symptoms at birth of CNS involvement were not found to significantly affect the onset of unilateral or bilateral SNHL (*p* > 0.05). Furthermore, none of these factors were found to significantly affect the onset of severe-to-profound SNHL (*p* > 0.05).

## 4. Discussion

The present study aims to identify potential predictive factors for SNHL development in patients with cCMV infection. Abnormalities identifiable in brain MRI and brain ultrasound are already-known clinical predictors of SNHL in cCMV infection [23]. The present study confirmed that the presence of abnormal findings in brain MRI in patients with symptomatic cCMV infection has a significant correlation with the onset of SNHL. In line with this result, Alarcon et al. [9,21] assessed a significant association with adverse neurodevelopmental outcomes, including hearing loss, and neuroimaging abnormalities analyzing the cranial imaging (including MRI) of 26 patients with symptomatic cCMV. In a recent systematic review focused on the predictive role of brain MRI on clinical outcomes in cCMV infection [24], the authors reported a correlation between MRI abnormalities and neurodevelopmental deficits, in particular with hearing loss. Compared to brain ultrasound, MRI can detect further cerebral anomalies, in both pre- and postnatal periods; it could represent a valid tool, complementary to ultrasound, in the diagnostic assessment of subjects with cCMV infection. In particular, the scoring system proposed by Alarcon et al. [21] was used for the analysis of MRI images; this classification allows for a four-level stratification, depending on the number and severity of the following brain abnormalities: intracerebral calcifications, ventriculomegaly, white matter changes, periventricular radiolucencies, pachygyria, lissencephaly, cortical dysplasia or atrophy, cerebellar abnormalities, hippocampal dysplasia, caudo-thalamic or paraventricular germinolytic cysts, and corpus callosum dysgenesis. These brain changes are typical signs of cCMV infection [25,26]. However, the pathophysiology of SNHL in cCMV infection is still not completely clear. Also, considering SNHL as a manifestation of virus-induced CNS abnormalities is still debated. Instead, available data in the literature suggest SNHL to be a consequence of the direct infection and damage of the inner ear structures [27,28], such as the loss of spiral ganglion neurons and the degeneration of the cochlear vasculature [27]. Furthermore, the susceptibility of the infant’s brain to viral infection, which is greater than the adult’s, can facilitate the onset of these viral-induced damages [29]. The marked CMV tropism for the inner ear has already been reported in the literature [30], even if the pathogenesis of the cochlear damage, the mechanism of viral spread, and the viral persistence in the inner ear have not been fully elucidated to date. Diffuse cytomegalic cells have been described in the inner ear, as well as in the brain, in the placenta, and other organs, suggesting the hematogenous spread of the viral infection [30]. Studies in guinea pigs have shown that the mid-apical gyrus is the most frequently affected region of the cochlea, whereas the basal gyrus is involved in more extensive lesions less frequently [31]. At the histopathological level, lesions in the inner ear have been predominantly found at the midscale in the endolymphatic compartment. It has been reported that CMV predominantly affects the stria vascularis and its marginal cells; this can further alter the regulation of the electrochemical balance of cochlear fluids, leading to a secondary degeneration of the neuroepithelium [28,30,32]. Other data in the literature reported the presence of CMV DNA in the inner ear fluid even for several years after congenital infection in asymptomatic subjects as well [33,34]. Such viral tropism could be the cause of progressive or late-onset hearing loss, possibly also due to subsequent viral reactivation. Recently, the quantification of CMV DNA by PCR on samples of inner ear fluid collected during cochlear implantation surgery has been proposed as a possible diagnostic approach for CMV-related hearing loss [35]. CMV may also affect Reissner’s membrane and the supporting cells of the organ of Corti [28,30]. In addition, further studies in guinea pigs also described a smaller number of neurons in the spiral ganglion and a predominantly monocytic inflammatory infiltrate in the spiral ganglion and the stria vascularis [27]. The inflammatory infiltrate, predominantly composed of T-CD8-activated lymphocytes and mainly found close to CMV-positive cells, can contribute to hair cell damage [28]. Furthermore, lymphocytes have also been detected in human histological examination within the cochlear nerve and in the spiral ganglia; it could be speculated that this inflammatory infiltrate could affect nerve conduction [28]. However, to this day, there is still no standardized imaging, even more so in newborns, that allows for the identification of the status of the cochlear cells and the condition of the microcirculation of the inner ear.

Unlike other data reported in the literature, this study did not show any significant correlation between abnormalities detected in brain ultrasound and the onset of SNHL. This result could be due to the small number of subjects included and the retrospective nature of the study. Also, Elkan Miller et al. [36] did not find any significant association between hearing loss and neuroimaging anomalies identified in prenatal ultrasound. Hence, it could be speculated that prenatal neuroimaging could not be sufficient to predict adverse neurodevelopmental outcomes. Furthermore, it has already been reported that brain ultrasound presents a low sensitivity in detecting gyral and myelinization anomalies compared to brain MRI [37]; this fact could represent another possible reason for the discrepancy in SNHL prediction between these two imaging methods.

In symptomatic infection, neurological signs or symptoms can often be detected at birth, especially in the case of identifiable alterations in brain imaging. The present study assessed a trend of significance regarding the increased risk of SNHL onset in subjects with symptomatic cCMV infection compared with asymptomatic infection. Neurological sequelae are present in 60–90% of symptomatic subjects; the presence of seizures, alterations in muscle tone, and microcephaly are frequently found in symptomatic patients, as described by de Juan Gallach et al. [38]. In the literature, a significant association has been described between (i) polymicrogyria, ventriculomegaly, calcifications, white matter alterations, and the development of epilepsy, and between (ii) polymicrogyria and psychomotor retardation in the group of symptomatic subjects [38]. Concerning isolated white matter alteration, no specific pattern of this lesion has been reported in the literature to correlate with long-term neurological prognosis or with the onset of hearing loss. Similarly, microcephaly has been reported to be the only certain indicator of severe neurological sequelae [38]. According to the data in the literature, the present study showed that signs and symptoms of CNS involvement at birth represented a factor significantly correlated with SNHL onset [9]. Pinninti et al. [39] have already reported, in 2016, that clinical CNS involvement (including microcephaly, seizures, lethargy/hypotonia) was significantly more associated with SNHL development compared with transient clinical findings (e.g., petechial rash) in a group of 160 infants with symptomatic cCMV infection. Therefore, the diagnostic work-up of newborns with cCMV infection should always include a complete neurological assessment to identify possible signs and/or symptoms of CNS involvement. A neurological exam could be even more crucial to guide clinical follow-up, especially when neuroimaging alterations have already been detected, in order to predict the onset of epilepsy and psychomotor delay.

Regarding the type of maternal CMV infection, it has been described that SNHL may be a consequence of maternal primary infection, transmitted vertically in any trimester of pregnancy [8,40,41], indicating that an audiological follow-up should be necessary in all cases of congenitally CMV infected infants. However, it has been estimated that maternal non-primary infection accounts for most cCMV infection cases worldwide [42], therefore causing SNHL in congenitally infected subjects [43]. In the present sample, no significant differences were found between maternal primary and non-primary infections in terms of SNHL development, occurrence of unilateral or bilateral hearing loss, and occurrence of severe-to-profound hearing loss. In Italy, there is still no routine prenatal screening for CMV in pregnancy [20]; hence, ascertaining the type of maternal infection and the time of in utero infection during gestation is often difficult in clinical practice. To the best of our knowledge, there are no effective interventions to prevent in utero CMV transmission to this date.

Concerning the timing of in utero infection, the present study identified that infections occurring during the first trimester are at a greater risk of developing SNHL than infections occurring in the second or third trimester, as per the data in the literature. Recently, Chebib et al. [44] reported that infection acquired during the first trimester represents an independent predictive factor of inner ear impairment. A previous study assessed that children infected during the first trimester of primary CMV infection were more likely to develop SNHL compared to those infected later in pregnancy [45]. Overall, first trimester cCMV infection during maternal primary infection seems to correlate with the most severe prognosis [46]. Embryogenesis studies focusing on the effects of in utero CMV infection reported that the type and extent of damages, and in particular brain sequelae, correlate with gestation; these viral-induced anomalies seem to be diverse throughout the pregnancy, depending on the variation of the susceptibility of embryo cells [47]. The higher risk of SNHL development in the case of in utero infection occurring during the first trimester compared to the others could be due to the higher vulnerability of the ear to the virus during the early period of gestation. In fact, the cochlear membranous labyrinth is formed between the third and tenth weeks of gestation, so early infection could provide a significantly higher risk of inner ear impairment, although a significant correlation between SNHL onset and time of infection during pregnancy has not been confirmed yet.

This study’s major limitations are the small sample size, its retrospective nature, and the absence of reliable viral load data at birth. Virus burden in blood and urine could represent a potential tool in the work-up of cCMV infection as a predictive factor of outcomes. It has been reported that patients with symptomatic cCMV infection show significantly higher blood and urine viral load mean values compared with those with asymptomatic infection [10,12]. Lanari et al. found statistically higher mean values of neonatal blood viral load in newborns with cCMV infection who developed sequelae (including hearing loss) than in those without adverse outcomes [12]; these data have been confirmed more recently by De Cuyper et al., even if without significant differences [23]. Boppana et al. showed a higher mean level of viral load in the urine of children with SNHL than the ones with normal hearing, with a significant difference in the group of asymptomatic patients but not in the symptomatic subjects [10]. Forner et al. [11] reported an increased risk of hearing loss with a blood viral burden ≥ 17,000 copies/mL in a group of 33 newborns with asymptomatic cCMV infection. Nonetheless, Forli et al. [48] did not find any significant association between mean viral load in the urine at birth with long-term sequelae, including hearing loss. Available data in the literature are currently contradictory and the role of viral burden as a predictive factor for hearing loss still needs to be clarified.

Currently, there are other potential predictive biomarkers for SNHL development tested in cCMV infection. Bioinformatic analyses applying next-generation sequencing explored the role of variants of CMV genotypes with no definitive results [49,50]. By applying whole-blood transcriptomics on 80 infants with cCMV infection, Ouellette et al. [51] identified a 16-gene classifier set associated with the development of SNHL with 92% accuracy, suggesting its potential value as a biomarker for SNHL. These findings advocate for a focus on the role of genome diversity as a predictive biomarker of SNHL in cCMV infection and suggest the need for further studies in order to define possible advantages of these bioinformatic techniques compared to the existing clinical and imaging predictive factors.

## 5. Conclusions

The present study showed an increased risk for SNHL development in patients with symptomatic cCMV infection. In particular, the presence of neurologic signs and/or symptoms at birth and the presence of detectable abnormalities in brain MRI were found to be predictive factors for SNHL; these features are in the line with the literature findings. In patients with symptomatic cCMV and, especially, presenting clinical signs or symptoms of CNS involvement at birth, brain MRI should be recommended as soon as possible, particularly if not yet performed during the fetal period. The use of a neuroimaging scale to classify the severity of brain abnormalities in MRI could help predict SNHL development.

Nonetheless, subjects with asymptomatic cCMV are still at risk of developing SNHL (also with a late onset), especially if the infection is acquired during the first gestational trimester. In our opinion, although there is still no universal neonatal screening protocol for cCMV, a strict audiological follow-up should be recommended for symptomatic and asymptomatic patients. Further studies involving a higher number of subjects and larger amounts of data on viral load are necessary. To improve the comparison among studies, it would be necessary to use uniform diagnostic criteria for cCMV infection, uniform definitions of symptomatic and asymptomatic infection, and to standardize the data on neonatal hearing screening. The current lack of reliable and comparable viral load levels might be explained by the poor accessibility of the data; appropriate improvements for future studies could be a major uniformity of the clinical use of blood and urine viral burden and of the time of the sample collection, as well as a standardization of the test across laboratories.

## Figures and Tables

**Table 1:** Characteristics of 50 subjects with congenital CMV infection.

	Cohort (n = 50, 100%)
Congenital CMV infection - asymptomatic - symptomatic	26 (52) 24 (48)
Gender - male - female	24 (48) 26 (52)
Maternal CMV infection - primary - non-primary	6 (12) 6 (12)
Time of in utero CMV infection - first trimester - second trimester - third trimester	12 (24) 2 (4) 4 (8)
SNHL - at birth - late-onset - unilateral - bilateral - severe-to-profound	38 (76) 14 (28) 6 (12) 8 (16) 20 (40) 24 (48)

CMV: Cytomegalovirus; SNHL: sensorineural hearing loss.

**Table 2:** Neuroimaging findings of subjects with symptomatic congenital CMV infection.

	Cohort (n = 24, 100%)
Brain ultrasound - ventricular dilatation - germinative cysts - lenticulostriate vasculopathy - occipital cystic lesions - pseudocyst	4 (16.7) 2 (8.3) 2 (8.3) 1 (4.2) 1 (4.2)
Brain MRI - white matter abnormalities - ventriculomegaly - periventricular radiolucency - temporal lobe lesions - intracerebral calcification - pachygyria - cortical dysplasia or atrophy - hippocampal dysplasia - brain atrophy	14 (58.3) 10 (41.7) 7 (29.2) 6 (25) 2 (8.3) 2 (8.3) 1 (4.2) 1 (4.2) 2 (8.3)

MRI: magnetic resonance imaging.

**Table 3:** Analysis of risk factors for the occurrence of sensorineural hearing loss in individuals with congenital CMV infection.

	SNHL	No SNHL	χ^2^ *p* Value	Univariate Analysis *p* Value
- Asymptomatic cCMV infection - Symptomatic cCMV infection	17/26 (65.4%) 21/24 (87.5%)	9/26 (34.6%) 3/24 (12.5%)	*p* = 0.067	*p* = 0.078
- Maternal primary CMV infection - Maternal non-primary CMV infection	5/6 (83.3%) 3/6 (50%)	1/6 (16.7%) 3/6 (50%)	*p* = 0.273	*p* = 0.239
- In utero infection during the first trimester - In utero infection during the second trimester - In utero infection during the third trimester	10/12 (83.3%) 1/2 (50%) 1/4 (25%)	2/12 (16.7%) 1/2 (50%) 3/4 (75%)	*p* = 0.087	*p* = 0.047 *
- In utero infection during the first trimester - In utero infection during the second and third trimesters	10/12 (83.3%) 2/6 (33.3%)	2/12 (16.7%) 4/6 (66.7%)	*p* = 0.057	*p* = 0.048 *
- Absence of signs and/or symptoms at birth of CNS involvement - Presence of signs and/or symptoms at birth of CNS involvement	19/30 (63.3%) 13/13 (100%)	11/30(36.7%) 0/13 (0%)	*p* = 0.009 *	*p* = 0.999
- Absence of systemic signs and/or symptoms and/or laboratory changes at birth due to cCMV infection - Presence of systemic signs and/or symptoms and/or laboratory changes at birth due to cCMV infection	22/30 (73.3%) 6/6 (100%)	8/30 (26.7%) 0/6 (0%)	*p* = 0.193	*p* = 0.999
- Male gender - Female gender	19/24 (79.2%) 19/26 (73.1%)	5/24 (20.8%) 7/26 (26.9%)	*p* = 0.614	*p* = 0.615

SNHL: sensorineural hearing loss; cCMV: congenital Cytomegalovirus; CMV: Cytomegalovirus; CNS: central nervous system. * Value of probability (*p* Value) of less than 0.05 were considered statistically significant.

## Data Availability

The original contributions presented in this study are included in the article; further inquiries can be directed to the corresponding author.
